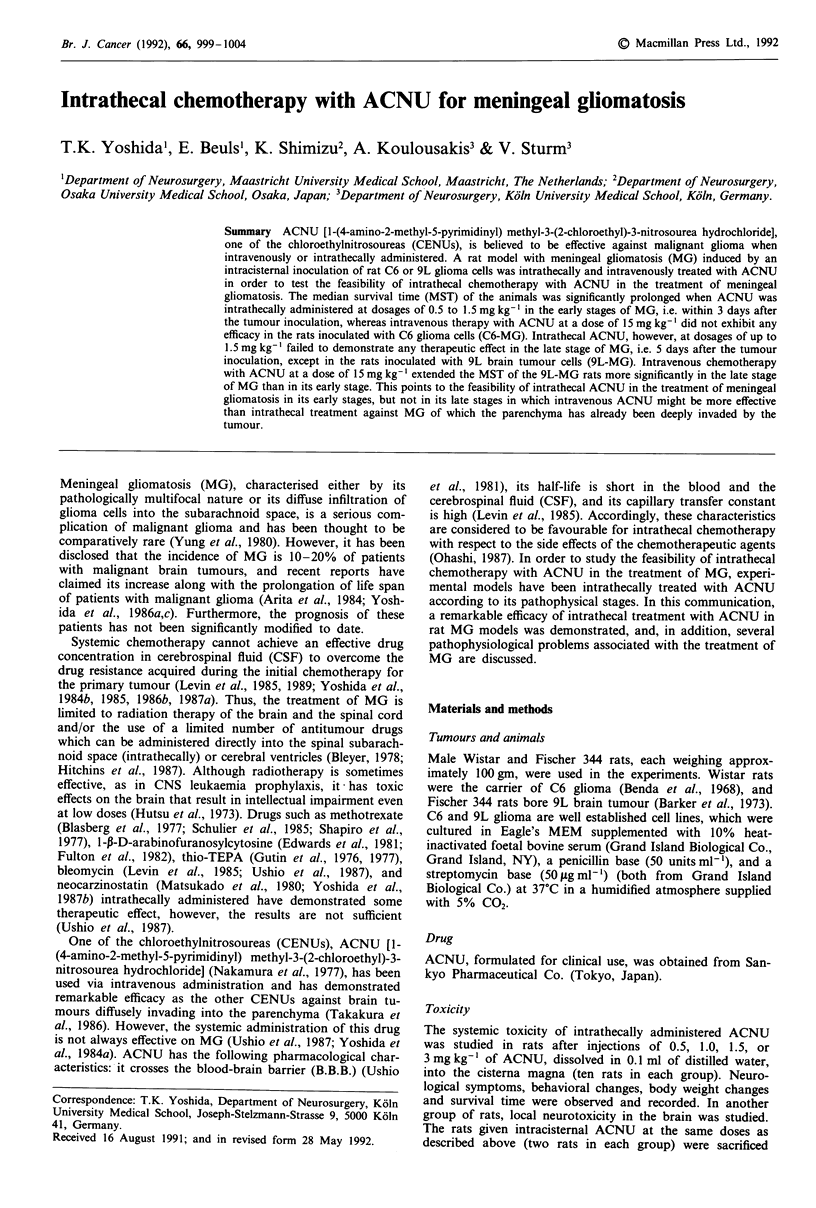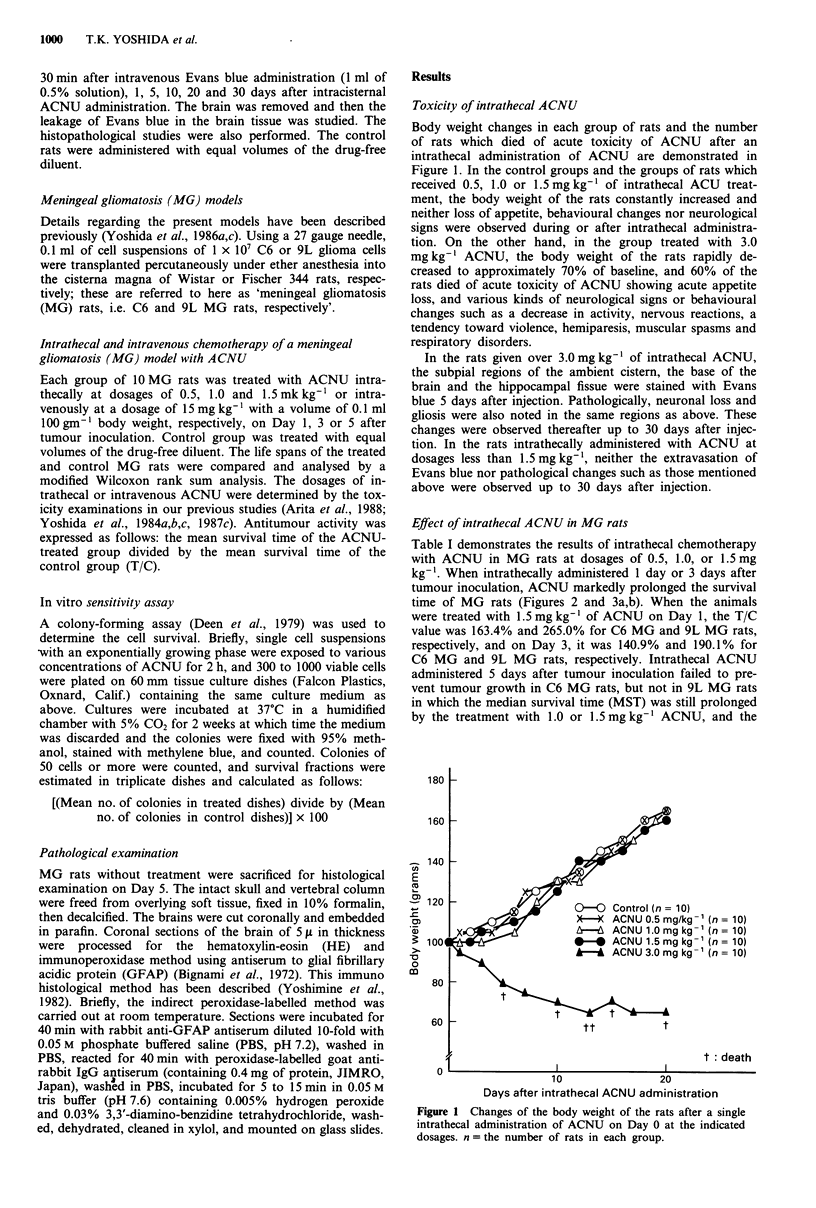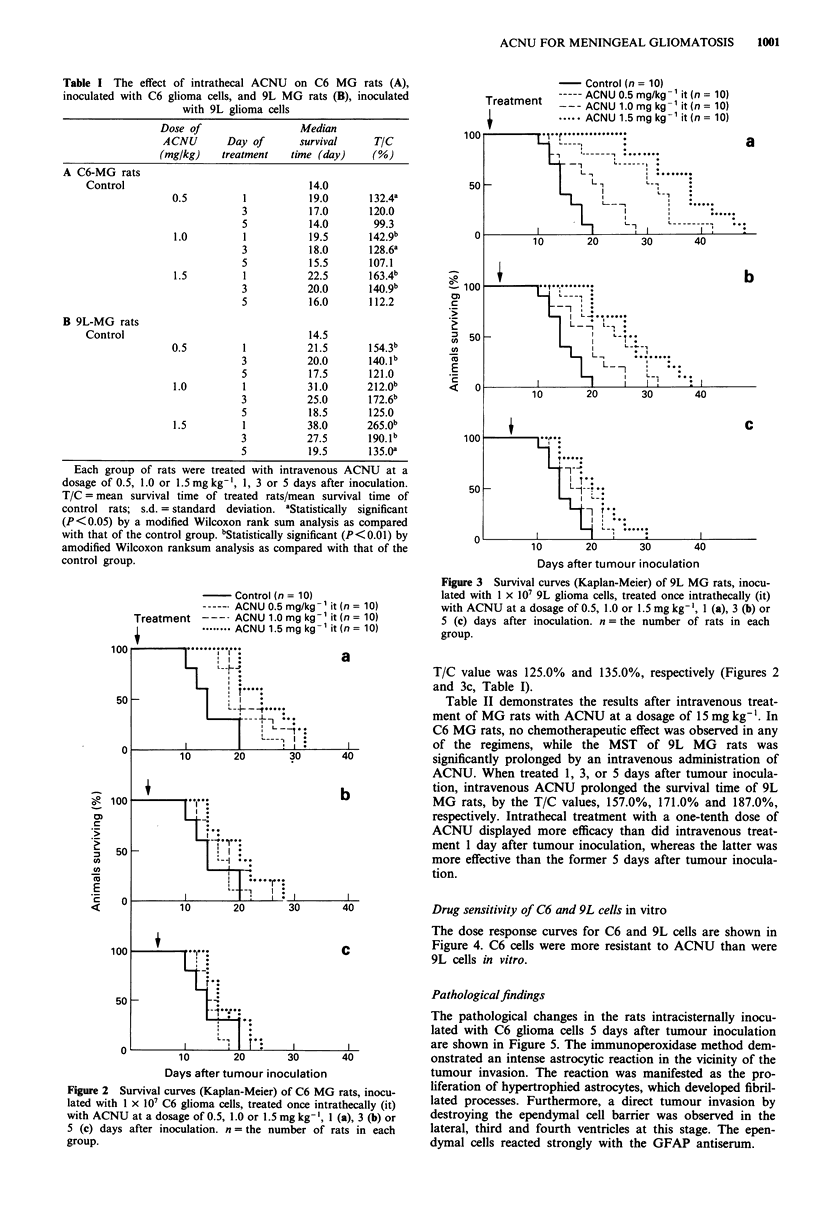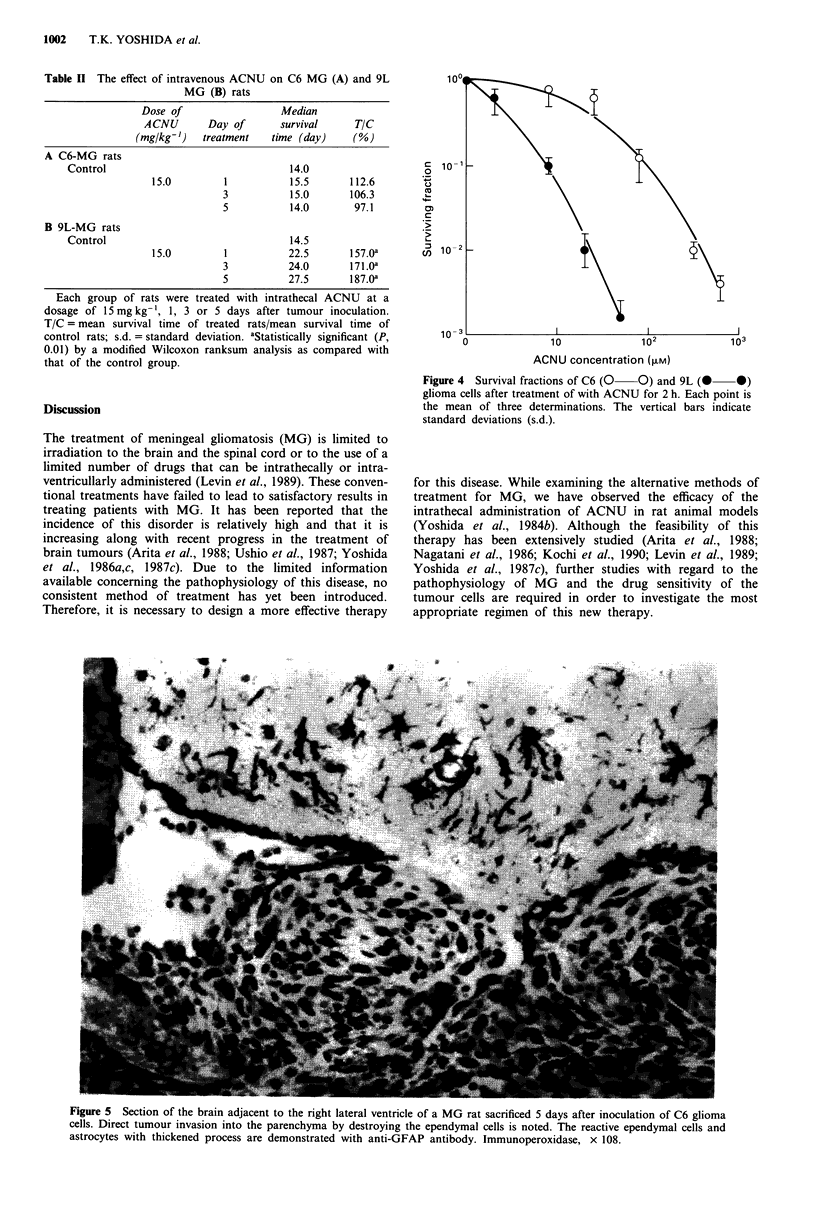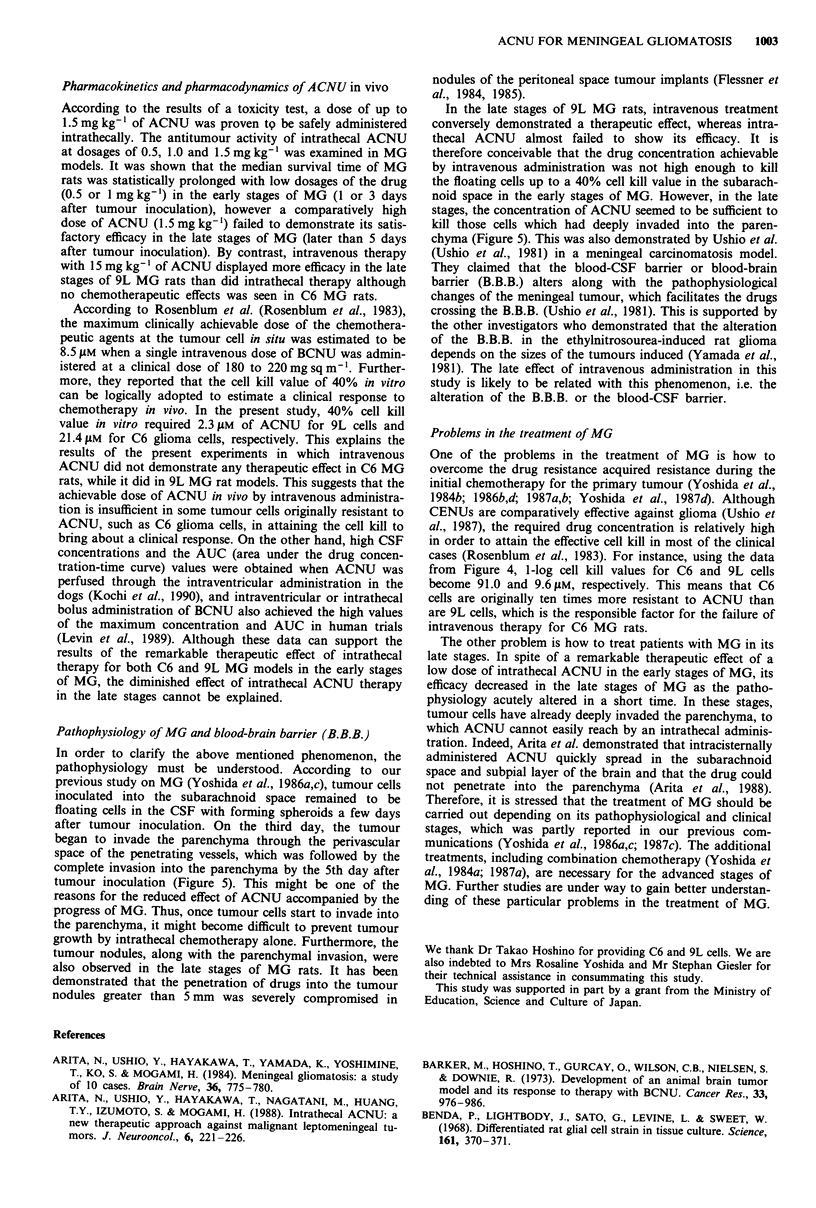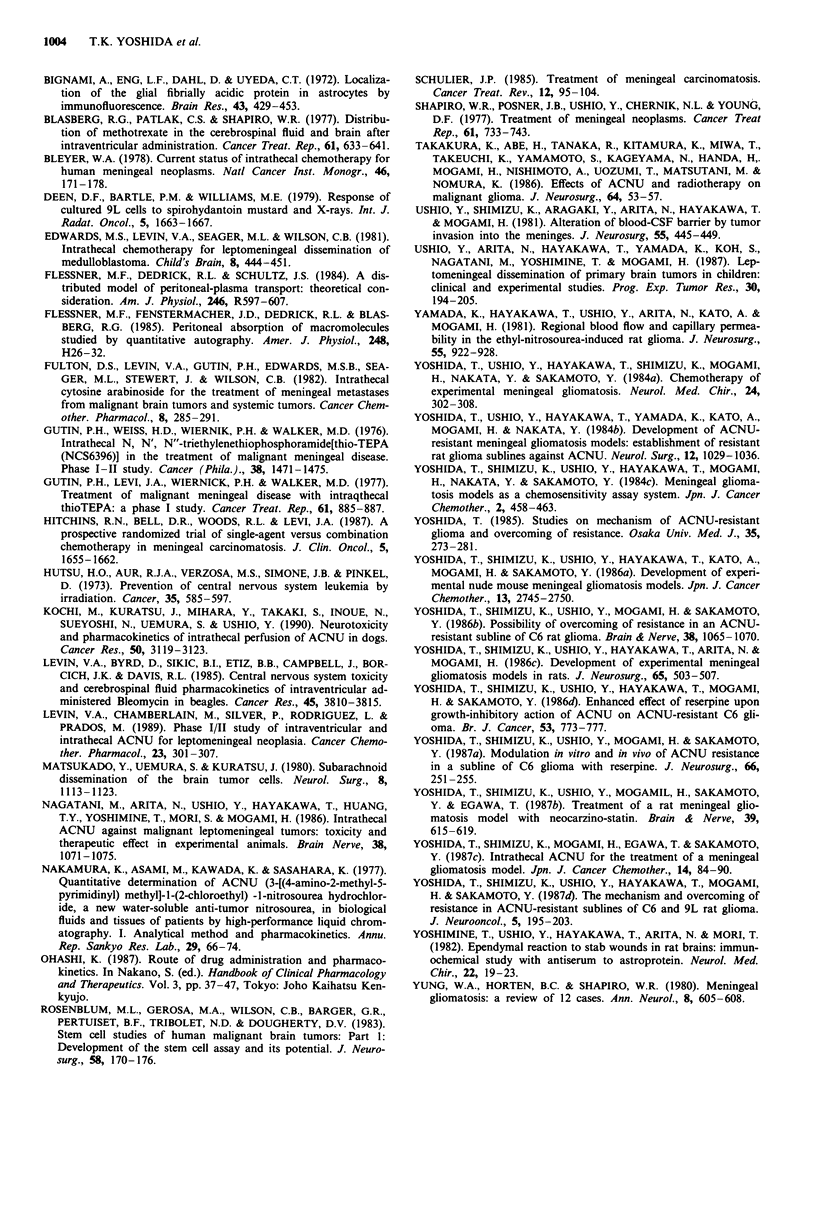# Intrathecal chemotherapy with ACNU for meningeal gliomatosis.

**DOI:** 10.1038/bjc.1992.401

**Published:** 1992-12

**Authors:** T. K. Yoshida, E. Beuls, K. Shimizu, A. Koulousakis, V. Sturm

**Affiliations:** Department of Neurosurgery, Maastricht University Medical School, The Netherlands.

## Abstract

**Images:**


*Br. J. Cancer* (1992), **66**, 999–1004 © Macmillan Press Ltd., 1992

### Intrathecal chemotherapy with ACNU for meningeal gliomatosis

T.K. Yoshida[1], E. Beuls[1], K. Shimizu[2], A. Koulousakis[3] & V. Sturm[3]

[1]*Department of Neurosurgery, Maastricht University Medical School, Maastricht, The Netherlands;* [2]*Department of Neurosurgery, Osaka University Medical School, Osaka, Japan;* [3]*Department of Neurosurgery, Köln University Medical School, Köln, Germany.*

**Summary** ACNU [1-(4-amino-2-methyl-5-pyrimidinyl) methyl-3-(2-chloroethyl)-3-nitrosourea hydrochloride], one of the chloroethylnitrosoureas (CENUs), is believed to be effective against malignant glioma when intravenously or intrathecally administered. A rat model with meningeal gliomatosis (MG) induced by an intracisternal inoculation of rat C6 or 9L glioma cells was intrathecally and intravenously treated with ACNU in order to test the feasibility of intrathecal chemotherapy with ACNU in the treatment of meningeal gliomatosis. The median survival time (MST) of the animals was significantly prolonged when ACNU was intrathecally administered at dosages of 0.5 to 1.5 mg $kg^{-1}$ in the early stages of MG, i.e. within 3 days after the tumour inoculation, whereas intravenous therapy with ACNU at a dose of 15 mg $kg^{-1}$ did not exhibit any efficacy in the rats inoculated with C6 glioma cells (C6-MG). Intrathecal ACNU, however, at dosages of up to 1.5 mg $kg^{-1}$ failed to demonstrate any therapeutic effect in the late stage of MG, i.e. 5 days after the tumour inoculation, except in the rats inoculated with 9L brain tumour cells (9L-MG). Intravenous chemotherapy with ACNU at a dose of 15 mg $kg^{-1}$ extended the MST of the 9L-MG rats more significantly in the late stage of MG than in its early stage. This points to the feasibility of intrathecal ACNU in the treatment of meningeal gliomatosis in its early stages, but not in its late stages in which intravenous ACNU might be more effective than intrathecal treatment against MG of which the parenchyma has already been deeply invaded by the tumour.

Meningeal gliomatosis (MG), characterised either by its pathologically multifocal nature or its diffuse infiltration of glioma cells into the subarachnoid space, is a serious complication of malignant glioma and has been thought to be comparatively rare (Yung *et al.*, 1980). However, it has been disclosed that the incidence of MG is 10–20% of patients with malignant brain tumours, and recent reports have claimed its increase along with the prolongation of life span of patients with malignant glioma (Arita *et al.*, 1984; Yoshida *et al.*, 1986*a,c*). Furthermore, the prognosis of these patients has not been significantly modified to date.

Systemic chemotherapy cannot achieve an effective drug concentration in cerebrospinal fluid (CSF) to overcome the drug resistance acquired during the initial chemotherapy for the primary tumour (Levin *et al.*, 1985, 1989; Yoshida *et al.*, 1984*b*, 1985, 1986*b*, 1987*a*). Thus, the treatment of MG is limited to radiation therapy of the brain and the spinal cord and/or the use of a limited number of antitumour drugs which can be administered directly into the spinal subarachnoid space (intrathecally) or cerebral ventricles (Bleyer, 1978; Hitchins *et al.*, 1987). Although radiotherapy is sometimes effective, as in CNS leukaemia prophylaxis, it has toxic effects on the brain that result in intellectual impairment even at low doses (Hutsu *et al.*, 1973). Drugs such as methotrexate (Blasberg *et al.*, 1977; Schulier *et al.*, 1985; Shapiro *et al.*, 1977), 1-β-D-arabinofuranosylcytosine (Edwards *et al.*, 1981; Fulton *et al.*, 1982), thio-TEPA (Gutin *et al.*, 1976, 1977), bleomycin (Levin *et al.*, 1985; Ushio *et al.*, 1987), and neocarzinostatin (Matsukado *et al.*, 1980; Yoshida *et al.*, 1987*b*) intrathecally administered have demonstrated some therapeutic effect, however, the results are not sufficient (Ushio *et al.*, 1987).

One of the chloroethylnitrosoureas (CENUs), ACNU [1-(4-amino-2-methyl-5-pyrimidinyl) methyl-3-(2-chloroethyl)-3-nitrosourea hydrochloride] (Nakamura *et al.*, 1977), has been used via intravenous administration and has demonstrated remarkable efficacy as the other CENUs against brain tumours diffusely invading into the parenchyma (Takakura *et al.*, 1986). However, the systemic administration of this drug is not always effective on MG (Ushio *et al.*, 1987; Yoshida *et al.*, 1984*a*). ACNU has the following pharmacological characteristics: it crosses the blood-brain barrier (B.B.B.) (Ushio *et al.*, 1981), its half-life is short in the blood and the cerebrospinal fluid (CSF), and its capillary transfer constant is high (Levin *et al.*, 1985). Accordingly, these characteristics are considered to be favourable for intrathecal chemotherapy with respect to the side effects of the chemotherapeutic agents (Ohashi, 1987). In order to study the feasibility of intrathecal chemotherapy with ACNU in the treatment of MG, experimental models have been intrathecally treated with ACNU according to its pathophysical stages. In this communication, a remarkable efficacy of intrathecal treatment with ACNU in rat MG models was demonstrated, and, in addition, several pathophysiological problems associated with the treatment of MG are discussed.

Correspondence: T.K. Yoshida, Department of Neurosurgery, Köln University Medical School, Joseph-Stelzmann-Strasse 9, 5000 Köln 41, Germany.

Received 16 August 1991; and in revised form 28 May 1992.

### Materials and methods

### *Tumours and animals*

Male Wistar and Fischer 344 rats, each weighing approximately 100 gm, were used in the experiments. Wistar rats were the carrier of C6 glioma (Benda *et al.*, 1968), and Fischer 344 rats bore 9L brain tumour (Barker *et al.*, 1973). C6 and 9L glioma are well established cell lines, which were cultured in Eagle's MEM supplemented with 10% heat-inactivated foetal bovine serum (Grand Island Biological Co., Grand Island, NY), a penicillin base (50 units $ml^{-1}$), and a streptomycin base (50 μg $ml^{-1}$) (both from Grand Island Biological Co.) at 37°C in a humidified atmosphere supplied with 5% $CO_2$.

### *Drug*

ACNU, formulated for clinical use, was obtained from Sankyo Pharmaceutical Co. (Tokyo, Japan).

### *Toxicity*

The systemic toxicity of intrathecally administered ACNU was studied in rats after injections of 0.5, 1.0, 1.5, or 3 mg $kg^{-1}$ of ACNU, dissolved in 0.1 ml of distilled water, into the cisterna magna (ten rats in each group). Neurological symptoms, behavioral changes, body weight changes and survival time were observed and recorded. In another group of rats, local neurotoxicity in the brain was studied. The rats given intracisternal ACNU at the same doses as described above (two rats in each group) were sacrificed

30 min after intravenous Evans blue administration (1 ml of 0.5% solution), 1, 5, 10, 20 and 30 days after intracisternal ACNU administration. The brain was removed and then the leakage of Evans blue in the brain tissue was studied. The histopathological studies were also performed. The control rats were administered with equal volumes of the drug-free diluent.

*Meningeal gliomatosis (MG) models*

Details regarding the present models have been described previously (Yoshida *et al.*, 1986*a,c*). Using a 27 gauge needle, 0.1 ml of cell suspensions of $1 \times 10^7$ C6 or 9L glioma cells were transplanted percutaneously under ether anesthesia into the cisterna magna of Wistar or Fischer 344 rats, respectively; these are referred to here as 'meningeal gliomatosis (MG) rats, i.e. C6 and 9L MG rats, respectively'.

*Intrathecal and intravenous chemotherapy of a meningeal gliomatosis (MG) model with ACNU*

Each group of 10 MG rats was treated with ACNU intrathecally at dosages of 0.5, 1.0 and 1.5 mk $kg^{-1}$ or intravenously at a dosage of 15 mg $kg^{-1}$ with a volume of 0.1 ml 100 $gm^{-1}$ body weight, respectively, on Day 1, 3 or 5 after tumour inoculation. Control group was treated with equal volumes of the drug-free diluent. The life spans of the treated and control MG rats were compared and analysed by a modified Wilcoxon rank sum analysis. The dosages of intrathecal or intravenous ACNU were determined by the toxicity examinations in our previous studies (Arita *et al.*, 1988; Yoshida *et al.*, 1984*a,b,c*, 1987*c*). Antitumour activity was expressed as follows: the mean survival time of the ACNU-treated group divided by the mean survival time of the control group (T/C).

In vitro *sensitivity assay*

A colony-forming assay (Deen *et al.*, 1979) was used to determine the cell survival. Briefly, single cell suspensions with an exponentially growing phase were exposed to various concentrations of ACNU for 2 h, and 300 to 1000 viable cells were plated on 60 mm tissue culture dishes (Falcon Plastics, Oxnard, Calif.) containing the same culture medium as above. Cultures were incubated at 37°C in a humidified chamber with 5% $CO_2$ for 2 weeks at which time the medium was discarded and the colonies were fixed with 95% methanol, stained with methylene blue, and counted. Colonies of 50 cells or more were counted, and survival fractions were estimated in triplicate dishes and calculated as follows:

[(Mean no. of colonies in treated dishes) divide by (Mean no. of colonies in control dishes)] × 100

*Pathological examination*

MG rats without treatment were sacrificed for histological examination on Day 5. The intact skull and vertebral column were freed from overlying soft tissue, fixed in 10% formalin, then decalcified. The brains were cut coronally and embedded in parafin. Coronal sections of the brain of 5 μ in thickness were processed for the hematoxylin-eosin (HE) and immunoperoxidase method using antiserum to glial fibrillary acidic protein (GFAP) (Bignami *et al.*, 1972). This immuno histological method has been described (Yoshimine *et al.*, 1982). Briefly, the indirect peroxidase-labelled method was carried out at room temperature. Sections were incubated for 40 min with rabbit anti-GFAP antiserum diluted 10-fold with 0.05 M phosphate buffered saline (PBS, pH 7.2), washed in PBS, reacted for 40 min with peroxidase-labelled goat anti-rabbit IgG antiserum (containing 0.4 mg of protein, JIMRO, Japan), washed in PBS, incubated for 5 to 15 min in 0.05 M tris buffer (pH 7.6) containing 0.005% hydrogen peroxide and 0.03% 3,3'-diamino-benzidine tetrahydrochloride, washed, dehydrated, cleaned in xylol, and mounted on glass slides.

### Results

*Toxicity of intrathecal ACNU*

Body weight changes in each group of rats and the number of rats which died of acute toxicity of ACNU after an intrathecal administration of ACNU are demonstrated in Figure 1. In the control groups and the groups of rats which received 0.5, 1.0 or 1.5 mg $kg^{-1}$ of intrathecal ACU treatment, the body weight of the rats constantly increased and neither loss of appetite, behavioural changes nor neurological signs were observed during or after intrathecal administration. On the other hand, in the group treated with 3.0 mg $kg^{-1}$ ACNU, the body weight of the rats rapidly decreased to approximately 70% of baseline, and 60% of the rats died of acute toxicity of ACNU showing acute appetite loss, and various kinds of neurological signs or behavioural changes such as a decrease in activity, nervous reactions, a tendency toward violence, hemiparesis, muscular spasms and respiratory disorders.

In the rats given over 3.0 mg $kg^{-1}$ of intrathecal ACNU, the subpial regions of the ambient cistern, the base of the brain and the hippocampal fissue were stained with Evans blue 5 days after injection. Pathologically, neuronal loss and gliosis were also noted in the same regions as above. These changes were observed thereafter up to 30 days after injection. In the rats intrathecally administered with ACNU at dosages less than 1.5 mg $kg^{-1}$, neither the extravasation of Evans blue nor pathological changes such as those mentioned above were observed up to 30 days after injection.

*Effect of intrathecal ACNU in MG rats*

Table I demonstrates the results of intrathecal chemotherapy with ACNU in MG rats at dosages of 0.5, 1.0, or 1.5 mg $kg^{-1}$. When intrathecally administered 1 day or 3 days after tumour inoculation, ACNU markedly prolonged the survival time of MG rats (Figures 2 and 3a,b). When the animals were treated with 1.5 mg $kg^{-1}$ of ACNU on Day 1, the T/C value was 163.4% and 265.0% for C6 MG and 9L MG rats, respectively, and on Day 3, it was 140.9% and 190.1% for C6 MG and 9L MG rats, respectively. Intrathecal ACNU administered 5 days after tumour inoculation failed to prevent tumour growth in C6 MG rats, but not in 9L MG rats in which the median survival time (MST) was still prolonged by the treatment with 1.0 or 1.5 mg $kg^{-1}$ ACNU, and the

**Figure 1** Changes of the body weight of the rats after a single intrathecal administration of ACNU on Day 0 at the indicated dosages. $n$ = the number of rats in each group.

**Table I** The effect of intrathecal ACNU on C6 MG rats (A), inoculated with C6 glioma cells, and 9L MG rats (B), inoculated with 9L glioma cells

| | *Dose of ACNU (mg/kg)* | *Day of treatment* | *Median survival time (day)* | *T/C (%)* |
|---|---|---|---|---|
| **A** C6-MG rats | | | | |
| Control | | | 14.0 | |
| | 0.5 | 1 | 19.0 | 132.4[a] |
| | | 3 | 17.0 | 120.0 |
| | | 5 | 14.0 | 99.3 |
| | 1.0 | 1 | 19.5 | 142.9[b] |
| | | 3 | 18.0 | 128.6[a] |
| | | 5 | 15.5 | 107.1 |
| | 1.5 | 1 | 22.5 | 163.4[b] |
| | | 3 | 20.0 | 140.9[b] |
| | | 5 | 16.0 | 112.2 |
| **B** 9L-MG rats | | | | |
| Control | | | 14.5 | |
| | 0.5 | 1 | 21.5 | 154.3[b] |
| | | 3 | 20.0 | 140.1[b] |
| | | 5 | 17.5 | 121.0 |
| | 1.0 | 1 | 31.0 | 212.0[b] |
| | | 3 | 25.0 | 172.6[b] |
| | | 5 | 18.5 | 125.0 |
| | 1.5 | 1 | 38.0 | 265.0[b] |
| | | 3 | 27.5 | 190.1[b] |
| | | 5 | 19.5 | 135.0[a] |

Each group of rats were treated with intravenous ACNU at a dosage of 0.5, 1.0 or 1.5 mg $kg^{-1}$, 1, 3 or 5 days after inoculation. T/C = mean survival time of treated rats/mean survival time of control rats; s.d. = standard deviation. [a]Statistically significant ($P < 0.05$) by a modified Wilcoxon rank sum analysis as compared with that of the control group. [b]Statistically significant ($P < 0.01$) by amodified Wilcoxon ranksum analysis as compared with that of the control group.

**Figure 2** Survival curves (Kaplan-Meier) of C6 MG rats, inoculated with 1 × $10^7$ C6 glioma cells, treated once intrathecally (it) with ACNU at a dosage of 0.5, 1.0 or 1.5 mg $kg^{-1}$, 1 (**a**), 3 (**b**) or 5 (**c**) days after inoculation. $n$ = the number of rats in each group.

**Figure 3** Survival curves (Kaplan-Meier) of 9L MG rats, inoculated with 1 × $10^7$ 9L glioma cells, treated once intrathecally (it) with ACNU at a dosage of 0.5, 1.0 or 1.5 mg $kg^{-1}$, 1 (**a**), 3 (**b**) or 5 (**c**) days after inoculation. $n$ = the number of rats in each group.

T/C value was 125.0% and 135.0%, respectively (Figures 2 and 3c, Table I).

Table II demonstrates the results after intravenous treatment of MG rats with ACNU at a dosage of 15 mg $kg^{-1}$. In C6 MG rats, no chemotherapeutic effect was observed in any of the regimens, while the MST of 9L MG rats was significantly prolonged by an intravenous administration of ACNU. When treated 1, 3, or 5 days after tumour inoculation, intravenous ACNU prolonged the survival time of 9L MG rats, by the T/C values, 157.0%, 171.0% and 187.0%, respectively. Intrathecal treatment with a one-tenth dose of ACNU displayed more efficacy than did intravenous treatment 1 day after tumour inoculation, whereas the latter was more effective than the former 5 days after tumour inoculation.

### *Drug sensitivity of C6 and 9L cells* in vitro

The dose response curves for C6 and 9L cells are shown in Figure 4. C6 cells were more resistant to ACNU than were 9L cells *in vitro*.

### *Pathological findings*

The pathological changes in the rats intracisternally inoculated with C6 glioma cells 5 days after tumour inoculation are shown in Figure 5. The immunoperoxidase method demonstrated an intense astrocytic reaction in the vicinity of the tumour invasion. The reaction was manifested as the proliferation of hypertrophied astrocytes, which developed fibrillated processes. Furthermore, a direct tumour invasion by destroying the ependymal cell barrier was observed in the lateral, third and fourth ventricles at this stage. The ependymal cells reacted strongly with the GFAP antiserum.

**Table II** The effect of intravenous ACNU on C6 MG (A) and 9L MG (B) rats

| | *Dose of ACNU (mg/kg⁻¹)* | *Day of treatment* | *Median survival time (day)* | *T/C (%)* |
|---|---|---|---|---|
| **A** C6-MG rats | | | | |
| Control | | | 14.0 | |
| | 15.0 | 1 | 15.5 | 112.6 |
| | | 3 | 15.0 | 106.3 |
| | | 5 | 14.0 | 97.1 |
| **B** 9L-MG rats | | | | |
| Control | | | 14.5 | |
| | 15.0 | 1 | 22.5 | 157.0[a] |
| | | 3 | 24.0 | 171.0[a] |
| | | 5 | 27.5 | 187.0[a] |

Each group of rats were treated with intrathecal ACNU at a dosage of 15 mg kg$^{-1}$, 1, 3 or 5 days after tumour inoculation. T/C = mean survival time of treated rats/mean survival time of control rats; s.d. = standard deviation. [a]Statistically significant (*P*, 0.01) by a modified Wilcoxon ranksum analysis as compared with that of the control group.

**Figure 4** Survival fractions of C6 (O——O) and 9L (●——●) glioma cells after treatment of with ACNU for 2 h. Each point is the mean of three determinations. The vertical bars indicate standard deviations (s.d.).

### Discussion

The treatment of meningeal gliomatosis (MG) is limited to irradiation to the brain and the spinal cord or to the use of a limited number of drugs that can be intrathecally or intraventricullarly administered (Levin *et al.*, 1989). These conventional treatments have failed to lead to satisfactory results in treating patients with MG. It has been reported that the incidence of this disorder is relatively high and that it is increasing along with recent progress in the treatment of brain tumours (Arita *et al.*, 1988; Ushio *et al.*, 1987; Yoshida *et al.*, 1986*a*,*c*, 1987*c*). Due to the limited information available concerning the pathophysiology of this disease, no consistent method of treatment has yet been introduced. Therefore, it is necessary to design a more effective therapy for this disease. While examining the alternative methods of treatment for MG, we have observed the efficacy of the intrathecal administration of ACNU in rat animal models (Yoshida *et al.*, 1984*b*). Although the feasibility of this therapy has been extensively studied (Arita *et al.*, 1988; Nagatani *et al.*, 1986; Kochi *et al.*, 1990; Levin *et al.*, 1989; Yoshida *et al.*, 1987*c*), further studies with regard to the pathophysiology of MG and the drug sensitivity of the tumour cells are required in order to investigate the most appropriate regimen of this new therapy.

**Figure 5** Section of the brain adjacent to the right lateral ventricle of a MG rat sacrificed 5 days after inoculation of C6 glioma cells. Direct tumour invasion into the parenchyma by destroying the ependymal cells is noted. The reactive ependymal cells and astrocytes with thickened process are demonstrated with anti-GFAP antibody. Immunoperoxidase, × 108.

*Pharmacokinetics and pharmacodynamics of ACNU* in vivo

According to the results of a toxicity test, a dose of up to $1.5\,mg\,kg^{-1}$ of ACNU was proven tọ be safely administered intrathecally. The antitumour activity of intrathecal ACNU at dosages of 0.5, 1.0 and $1.5\,mg\,kg^{-1}$ was examined in MG models. It was shown that the median survival time of MG rats was statistically prolonged with low dosages of the drug (0.5 or $1\,mg\,kg^{-1}$) in the early stages of MG (1 or 3 days after tumour inoculation), however a comparatively high dose of ACNU ($1.5\,mg\,kg^{-1}$) failed to demonstrate its satisfactory efficacy in the late stages of MG (later than 5 days after tumour inoculation). By contrast, intravenous therapy with $15\,mg\,kg^{-1}$ of ACNU displayed more efficacy in the late stages of 9L MG rats than did intrathecal therapy although no chemotherapeutic effects was seen in C6 MG rats.

According to Rosenblum *et al.* (Rosenblum *et al.*, 1983), the maximum clinically achievable dose of the chemotherapeutic agents at the tumour cell *in situ* was estimated to be $8.5\,\mu M$ when a single intravenous dose of BCNU was administered at a clinical dose of 180 to $220\,mg\,sq\,m^{-1}$. Furthermore, they reported that the cell kill value of 40% *in vitro* can be logically adopted to estimate a clinical response to chemotherapy *in vivo*. In the present study, 40% cell kill value *in vitro* required $2.3\,\mu M$ of ACNU for 9L cells and $21.4\,\mu M$ for C6 glioma cells, respectively. This explains the results of the present experiments in which intravenous ACNU did not demonstrate any therapeutic effect in C6 MG rats, while it did in 9L MG rat models. This suggests that the achievable dose of ACNU *in vivo* by intravenous administration is insufficient in some tumour cells originally resistant to ACNU, such as C6 glioma cells, in attaining the cell kill to bring about a clinical response. On the other hand, high CSF concentrations and the AUC (area under the drug concentration-time curve) values were obtained when ACNU was perfused through the intraventricular administration in the dogs (Kochi *et al.*, 1990), and intraventricular or intrathecal bolus administration of BCNU also achieved the high values of the maximum concentration and AUC in human trials (Levin *et al.*, 1989). Although these data can support the results of the remarkable therapeutic effect of intrathecal therapy for both C6 and 9L MG models in the early stages of MG, the diminished effect of intrathecal ACNU therapy in the late stages cannot be explained.

*Pathophysiology of MG and blood-brain barrier (B.B.B.)*

In order to clarify the above mentioned phenomenon, the pathophysiology must be understood. According to our previous study on MG (Yoshida *et al.*, 1986*a,c*), tumour cells inoculated into the subarachnoid space remained to be floating cells in the CSF with forming spheroids a few days after tumour inoculation. On the third day, the tumour began to invade the parenchyma through the perivascular space of the penetrating vessels, which was followed by the complete invasion into the parenchyma by the 5th day after tumour inoculation (Figure 5). This might be one of the reasons for the reduced effect of ACNU accompanied by the progress of MG. Thus, once tumour cells start to invade into the parenchyma, it might become difficult to prevent tumour growth by intrathecal chemotherapy alone. Furthermore, the tumour nodules, along with the parenchymal invasion, were also observed in the late stages of MG rats. It has been demonstrated that the penetration of drugs into the tumour nodules greater than 5 mm was severely compromised in nodules of the peritoneal space tumour implants (Flessner *et al.*, 1984, 1985).

In the late stages of 9L MG rats, intravenous treatment conversely demonstrated a therapeutic effect, whereas intrathecal ACNU almost failed to show its efficacy. It is therefore conceivable that the drug concentration achievable by intravenous administration was not high enough to kill the floating cells up to a 40% cell kill value in the subarachnoid space in the early stages of MG. However, in the late stages, the concentration of ACNU seemed to be sufficient to kill those cells which had deeply invaded into the parenchyma (Figure 5). This was also demonstrated by Ushio *et al.* (Ushio *et al.*, 1981) in a meningeal carcinomatosis model. They claimed that the blood-CSF barrier or blood-brain barrier (B.B.B.) alters along with the pathophysiological changes of the meningeal tumour, which facilitates the drugs crossing the B.B.B. (Ushio *et al.*, 1981). This is supported by the other investigators who demonstrated that the alteration of the B.B.B. in the ethylnitrosourea-induced rat glioma depends on the sizes of the tumours induced (Yamada *et al.*, 1981). The late effect of intravenous administration in this study is likely to be related with this phenomenon, i.e. the alteration of the B.B.B. or the blood-CSF barrier.

*Problems in the treatment of MG*

One of the problems in the treatment of MG is how to overcome the drug resistance acquired resistance during the initial chemotherapy for the primary tumour (Yoshida *et al.*, 1984*b*; 1986*b,d*; 1987*a,b*; Yoshida *et al.*, 1987*d*). Although CENUs are comparatively effective against glioma (Ushio *et al.*, 1987), the required drug concentration is relatively high in order to attain the effective cell kill in most of the clinical cases (Rosenblum *et al.*, 1983). For instance, using the data from Figure 4, 1-log cell kill values for C6 and 9L cells become 91.0 and $9.6\,\mu M$, respectively. This means that C6 cells are originally ten times more resistant to ACNU than are 9L cells, which is the responsible factor for the failure of intravenous therapy for C6 MG rats.

The other problem is how to treat patients with MG in its late stages. In spite of a remarkable therapeutic effect of a low dose of intrathecal ACNU in the early stages of MG, its efficacy decreased in the late stages of MG as the pathophysiology acutely altered in a short time. In these stages, tumour cells have already deeply invaded the parenchyma, to which ACNU cannot easily reach by an intrathecal administration. Indeed, Arita *et al.* demonstrated that intracisternally administered ACNU quickly spread in the subarachnoid space and subpial layer of the brain and that the drug could not penetrate into the parenchyma (Arita *et al.*, 1988). Therefore, it is stressed that the treatment of MG should be carried out depending on its pathophysiological and clinical stages, which was partly reported in our previous communications (Yoshida *et al.*, 1986*a,c*; 1987*c*). The additional treatments, including combination chemotherapy (Yoshida *et al.*, 1984*a*; 1987*a*), are necessary for the advanced stages of MG. Further studies are under way to gain better understanding of these particular problems in the treatment of MG.

We thank Dr Takao Hoshino for providing C6 and 9L cells. We are also indebted to Mrs Rosaline Yoshida and Mr Stephan Giesler for their technical assistance in consummating this study.

This study was supported in part by a grant from the Ministry of Education, Science and Culture of Japan.

**References**

ARITA, N., USHIO, Y., HAYAKAWA, T., YAMADA, K., YOSHIMINE, T., KO, S. & MOGAMI, H. (1984). Meningeal gliomatosis: a study of 10 cases. *Brain Nerve*, **36**, 775–780.

ARITA, N., USHIO, Y., HAYAKAWA, T., NAGATANI, M., HUANG, T.Y., IZUMOTO, S. & MOGAMI, H. (1988). Intrathecal ACNU: a new therapeutic approach against malignant leptomeningeal tumors. *J. Neurooncol.*, **6**, 221–226.

BARKER, M., HOSHINO, T., GURCAY, O., WILSON, C.B., NIELSEN, S. & DOWNIE, R. (1973). Development of an animal brain tumor model and its response to therapy with BCNU. *Cancer Res.*, **33**, 976–986.

BENDA, P., LIGHTBODY, J., SATO, G., LEVINE, L. & SWEET, W. (1968). Differentiated rat glial cell strain in tissue culture. *Science*, **161**, 370–371.

BIGNAMI, A., ENG, L.F., DAHL, D. & UYEDA, C.T. (1972). Localization of the glial fibrially acidic protein in astrocytes by immunofluorescence. *Brain Res.*, **43**, 429–453.

BLASBERG, R.G., PATLAK, C.S. & SHAPIRO, W.R. (1977). Distribution of methotrexate in the cerebrospinal fluid and brain after intraventricular administration. *Cancer Treat. Rep.*, **61**, 633–641.

BLEYER, W.A. (1978). Current status of intrathecal chemotherapy for human meningeal neoplasms. *Natl Cancer Inst. Monogr.*, **46**, 171–178.

DEEN, D.F., BARTLE, P.M. & WILLIAMS, M.E. (1979). Response of cultured 9L cells to spirohydantoin mustard and X-rays. *Int. J. Radat. Oncol.*, **5**, 1663–1667.

EDWARDS, M.S., LEVIN, V.A., SEAGER, M.L. & WILSON, C.B. (1981). Intrathecal chemotherapy for leptomeningeal dissemination of medulloblastoma. *Child's Brain*, **8**, 444–451.

FLESSNER, M.F., DEDRICK, R.L. & SCHULTZ, J.S. (1984). A distributed model of peritoneal-plasma transport: theoretical consideration. *Am. J. Physiol.*, **246**, R597–607.

FLESSNER, M.F., FENSTERMACHER, J.D., DEDRICK, R.L. & BLASBERG, R.G. (1985). Peritoneal absorption of macromolecules studied by quantitative autography. *Amer. J. Physiol.*, **248**, H26–32.

FULTON, D.S., LEVIN, V.A., GUTIN, P.H., EDWARDS, M.S.B., SEAGER, M.L., STEWERT, J. & WILSON, C.B. (1982). Intrathecal cytosine arabinoside for the treatment of meningeal metastases from malignant brain tumors and systemic tumors. *Cancer Chemother. Pharmacol.*, **8**, 285–291.

GUTIN, P.H., WEISS, H.D., WIERNIK, P.H. & WALKER, M.D. (1976). Intrathecal N, N', N''-triethylenethiophosphoramide[thio-TEPA (NCS6396)] in the treatment of malignant meningeal disease. Phase I–II study. *Cancer (Phila.)*, **38**, 1471–1475.

GUTIN, P.H., LEVI, J.A., WIERNICK, P.H. & WALKER, M.D. (1977). Treatment of malignant meningeal disease with intraqthecal thioTEPA: a phase I study. *Cancer Treat. Rep.*, **61**, 885–887.

HITCHINS, R.N., BELL, D.R., WOODS, R.L. & LEVI, J.A. (1987). A prospective randomized trial of single-agent versus combination chemotherapy in meningeal carcinomatosis. *J. Clin. Oncol.*, **5**, 1655–1662.

HUTSU, H.O., AUR, R.J.A., VERZOSA, M.S., SIMONE, J.B. & PINKEL, D. (1973). Prevention of central nervous system leukemia by irradiation. *Cancer*, **35**, 585–597.

KOCHI, M., KURATSU, J., MIHARA, Y., TAKAKI, S., INOUE, N., SUEYOSHI, N., UEMURA, S. & USHIO, Y. (1990). Neurotoxicity and pharmacokinetics of intrathecal perfusion of ACNU in dogs. *Cancer Res.*, **50**, 3119–3123.

LEVIN, V.A., BYRD, D., SIKIC, B.I., ETIZ, B.B., CAMPBELL, J., BORCICH, J.K. & DAVIS, R.L. (1985). Central nervous system toxicity and cerebrospinal fluid pharmacokinetics of intraventricular administered Bleomycin in beagles. *Cancer Res.*, **45**, 3810–3815.

LEVIN, V.A., CHAMBERLAIN, M., SILVER, P., RODRIGUEZ, L. & PRADOS, M. (1989). Phase I/II study of intraventricular and intrathecal ACNU for leptomeningeal neoplasia. *Cancer Chemother. Pharmacol.*, **23**, 301–307.

MATSUKADO, Y., UEMURA, S. & KURATSU, J. (1980). Subarachnoid dissemination of the brain tumor cells. *Neurol. Surg.*, **8**, 1113–1123.

NAGATANI, M., ARITA, N., USHIO, Y., HAYAKAWA, T., HUANG, T.Y., YOSHIMINE, T., MORI, S. & MOGAMI, H. (1986). Intrathecal ACNU against malignant leptomeningeal tumors: toxicity and therapeutic effect in experimental animals. *Brain Nerve*, **38**, 1071–1075.

NAKAMURA, K., ASAMI, M., KAWADA, K. & SASAHARA, K. (1977). Quantitative determination of ACNU (3-[(4-amino-2-methyl-5-pyrimidinyl) methyl]-1-(2-chloroethyl) -1-nitrosourea hydrochloride, a new water-soluble anti-tumor nitrosourea, in biological fluids and tissues of patients by high-performance liquid chromatography. I. Analytical method and pharmacokinetics. *Annu. Rep. Sankyo Res. Lab.*, **29**, 66–74.

OHASHI, K. (1987). Route of drug administration and pharmacokinetics. In Nakano, S. (ed.). *Handbook of Clinical Pharmacology and Therapeutics*. Vol. 3, pp. 37–47, Tokyo: Joho Kaihatsu Kenkyujo.

ROSENBLUM, M.L., GEROSA, M.A., WILSON, C.B., BARGER, G.R., PERTUISET, B.F., TRIBOLET, N.D. & DOUGHERTY, D.V. (1983). Stem cell studies of human malignant brain tumors: Part 1: Development of the stem cell assay and its potential. *J. Neurosurg.*, **58**, 170–176.

SCHULIER, J.P. (1985). Treatment of meningeal carcinomatosis. *Cancer Treat. Rev.*, **12**, 95–104.

SHAPIRO, W.R., POSNER, J.B., USHIO, Y., CHERNIK, N.L. & YOUNG, D.F. (1977). Treatment of meningeal neoplasms. *Cancer Treat Rep.*, **61**, 733–743.

TAKAKURA, K., ABE, H., TANAKA, R., KITAMURA, K., MIWA, T., TAKEUCHI, K., YAMAMOTO, S., KAGEYAMA, N., HANDA, H,. MOGAMI, H., NISHIMOTO, A., UOZUMI, T., MATSUTANI, M. & NOMURA, K. (1986). Effects of ACNU and radiotherapy on malignant glioma. *J. Neurosurg.*, **64**, 53–57.

USHIO, Y., SHIMIZU, K., ARAGAKI, Y., ARITA, N., HAYAKAWA, T. & MOGAMI, H. (1981). Alteration of blood-CSF barrier by tumor invasion into the meninges. *J. Neurosurg*, **55**, 445–449.

USHIO, Y., ARITA, N., HAYAKAWA, T., YAMADA, K., KOH, S., NAGATANI, M., YOSHIMINE, T. & MOGAMI, H. (1987). Leptomeningeal dissemination of primary brain tumors in children: clinical and experimental studies. *Prog. Exp. Tumor Res.*, **30**, 194–205.

YAMADA, K., HAYAKAWA, T., USHIO, Y., ARITA, N., KATO, A. & MOGAMI, H. (1981). Regional blood flow and capillary permeability in the ethyl-nitrosourea-induced rat glioma. *J. Neurosurg.*, **55**, 922–928.

YOSHIDA, T., USHIO, Y., HAYAKAWA, T., SHIMIZU, K., MOGAMI, H., NAKATA, Y. & SAKAMOTO, Y. (1984*a*). Chemotherapy of experimental meningeal gliomatosis. *Neurol. Med. Chir.*, **24**, 302–308.

YOSHIDA, T., USHIO, Y., HAYAKAWA, T., YAMADA, K., KATO, A., MOGAMI, H. & NAKATA, Y. (1984*b*). Development of ACNU-resistant meningeal gliomatosis models: establishment of resistant rat glioma sublines against ACNU. *Neurol. Surg.*, **12**, 1029–1036.

YOSHIDA, T., SHIMIZU, K., USHIO, Y., HAYAKAWA, T., MOGAMI, H., NAKATA, Y. & SAKAMOTO, Y. (1984*c*). Meningeal gliomatosis models as a chemosensitivity assay system. *Jpn. J. Cancer Chemother.*, **2**, 458–463.

YOSHIDA, T. (1985). Studies on mechanism of ACNU-resistant glioma and overcoming of resistance. *Osaka Univ. Med. J.*, **35**, 273–281.

YOSHIDA, T., SHIMIZU, K., USHIO, Y., HAYAKAWA, T., KATO, A., MOGAMI, H. & SAKAMOTO, Y. (1986*a*). Development of experimental nude mouse meningeal gliomatosis models. *Jpn. J. Cancer Chemother.*, **13**, 2745–2750.

YOSHIDA, T., SHIMIZU, K., USHIO, Y., MOGAMI, H. & SAKAMOTO, Y. (1986*b*). Possibility of overcoming of resistance in an ACNU-resistant subline of C6 rat glioma. *Brain & Nerve*, **38**, 1065–1070.

YOSHIDA, T., SHIMIZU, K., USHIO, Y., HAYAKAWA, T., ARITA, N. & MOGAMI, H. (1986*c*). Development of experimental meningeal gliomatosis models in rats. *J. Neurosurg.*, **65**, 503–507.

YOSHIDA, T., SHIMIZU, K., USHIO, Y., HAYAKAWA, T., MOGAMI, H. & SAKAMOTO, Y. (1986*d*). Enhanced effect of reserpine upon growth-inhibitory action of ACNU on ACNU-resistant C6 glioma. *Br. J. Cancer*, **53**, 773–777.

YOSHIDA, T., SHIMIZU, K., USHIO, Y., MOGAMI, H. & SAKAMOTO, Y. (1987*a*). Modulation *in vitro* and *in vivo* of ACNU resistance in a subline of C6 glioma with reserpine. *J. Neurosurg.*, **66**, 251–255.

YOSHIDA, T., SHIMIZU, K., USHIO, Y., MOGAMIL, H., SAKAMOTO, Y. & EGAWA, T. (1987*b*). Treatment of a rat meningeal gliomatosis model with neocarzino-statin. *Brain & Nerve*, **39**, 615–619.

YOSHIDA, T., SHIMIZU, K., MOGAMI, H., EGAWA, T. & SAKAMOTO, Y. (1987*c*). Intrathecal ACNU for the treatment of a meningeal gliomatosis model. *Jpn. J. Cancer Chemother.*, **14**, 84–90.

YOSHIDA, T., SHIMIZU, K., USHIO, Y., HAYAKAWA, T., MOGAMI, H. & SAKAMOTO, Y. (1987*d*). The mechanism and overcoming of resistance in ACNU-resistant sublines of C6 and 9L rat glioma. *J. Neurooncol.*, **5**, 195–203.

YOSHIMINE, T., USHIO, Y., HAYAKAWA, T., ARITA, N. & MORI, T. (1982). Ependymal reaction to stab wounds in rat brains: immunochemical study with antiserum to astroprotein. *Neurol. Med. Chir.*, **22**, 19–23.

YUNG, W.A., HORTEN, B.C. & SHAPIRO, W.R. (1980). Meningeal gliomatosis: a review of 12 cases. *Ann. Neurol.*, **8**, 605–608.